# Evaluation of environmental bacterial communities as a factor affecting the growth of duckweed *Lemna minor*

**DOI:** 10.1186/s13068-017-0746-8

**Published:** 2017-03-10

**Authors:** Hidehiro Ishizawa, Masashi Kuroda, Masaaki Morikawa, Michihiko Ike

**Affiliations:** 1 Division of Sustainable Energy and Environmental Engineering, Graduate School of Engineering, Osaka University, 2-1 Yamadaoka, Suita, Osaka, 565-0871 Japan; 2 Division of Biosphere Science, Graduate School of Environmental Science, Hokkaido University, N10-W5, Kita-ku, Sapporo, 060-0810 Japan

**Keywords:** Duckweed, Plant growth-promoting bacteria, Plant growth-inhibiting bacteria, Biomass production

## Abstract

**Background:**

Duckweed (family *Lemnaceae*) has recently been recognized as an ideal biomass feedstock for biofuel production due to its rapid growth and high starch content, which inspired interest in improving their productivity. Since microbes that co-exist with plants are known to have significant effects on their growth according to the previous studies for terrestrial plants, this study has attempted to understand the plant–microbial interactions of a duckweed, *Lemna minor*, focusing on the growth promotion/inhibition effects so as to assess the possibility of accelerated duckweed production by modifying co-existing bacterial community.

**Results:**

Co-cultivation of aseptic *L. minor* and bacterial communities collected from various aquatic environments resulted in changes in duckweed growth ranging from −24 to +14% compared to aseptic control. A number of bacterial strains were isolated from both growth-promoting and growth-inhibitory communities, and examined for their co-existing effects on duckweed growth. Irrespective of the source, each strain showed promotive, inhibitory, or neutral effects when individually co-cultured with *L. minor*. To further analyze the interactions among these bacterial strains in a community, binary combinations of promotive and inhibitory strains were co-cultured with aseptic *L. minor*, resulting in that combinations of promotive–promotive or inhibitory–inhibitory strains generally showed effects similar to those of individual strains. However, combinations of promotive–inhibitory strains tended to show inhibitory effects while only *Aquitalea magnusonii* H3 exerted its plant growth-promoting effect in all combinations tested.

**Conclusion:**

Significant change in biomass production was observed when duckweed was co-cultivated with environmental bacterial communities. Promotive, neutral, and inhibitory bacteria in the community would synergistically determine the effects. The results indicate the possibility of improving duckweed biomass production via regulation of co-existing bacterial communities.

**Electronic supplementary material:**

The online version of this article (doi:10.1186/s13068-017-0746-8) contains supplementary material, which is available to authorized users.

## Background

Duckweed is a tiny floating aquatic plant that is characterized by a rapid growth, high tolerance to polluted water, global distribution, and high starch content [1]. For decades, duckweed was considered as an industrially versatile plant that could be used for animal feed [2, 3], organic fertilizer [4], and chemical toxicity tests [5, 6]. In recent years, duckweed has been recognized as an ideal feedstock for biofuel production, because their soft and starch-rich biomass enables larger yield of fuel ethanol, butanol, and biogas [7, 8]. Xu et al. [9] calculated that bioethanol production from duckweed is 1.5 times greater than that from maize, when considering all parts of the cultivation and fermentation processes. Further, since duckweed can efficiently remove nitrogen, phosphorus, and heavy metals from water during growth, it has also been used in low-cost and low-energy wastewater treatment systems [10–12]. Thus, co-beneficial systems that combine biofuel production and water purification using duckweed have been proposed [13].

The attractive features of duckweed as a biomass resource have inspired interest in improving their productivity via selection of species/strains with higher growth rates [14] and optimizing the design and operational parameters, such as harvest period and water depth, of cultivation systems [15–17]. The effects of nutrient strength [18, 19], light intensity, photoperiod [20], and temperature [19] on duckweed growth and starch accumulation have also been examined to improve the production.

In addition, microbes that co-exist with duckweed are believed to have significant effects on growth in natural cultivation systems. In the terrestrial sphere, plants are widely recognized to develop intimate interactions with microbes that are critical for their growth or survival [21]. Some symbiotic bacteria called plant growth-promoting bacteria (PGPB) are known to enhance host plant growth by increasing nutrient acquisition or alleviating biotic and abiotic stresses [22]. In the last few decades, considerable efforts have been dedicated to isolate and characterize PGPB for important terrestrial agricrops, and it is clear that an extremely wide range of the plants harbor beneficial bacteria such as PGPB.

Although there have been several studies to understand plant-associated microbial communities and to engineer them for optimal production of crops [23, 24], such studies on aquatic plants, including duckweed, have just started lately [25]. Crump et al. [26], Xie et al. [27], and Matsuzawa et al. [28] have recently found that aquatic plants, including duckweed, also harbor diverse and specific bacterial communities. Yamaga et al. [29] isolated the first PGPB recognized to promote duckweed growth in a sterile synthetic medium. Another bacterium was recently found to promote the growth of the duckweed *Lemna minor* in a medium containing chromium [30]. These confirmed that bacteria living with duckweed can exert significant effects on host plant growth, similar to those seen in terrestrial crops. Thus, extended studies on duckweed–microbe interactions, especially those affecting duckweed growth, are needed to realize efficient and sustainable cultivation of duckweed species utilizing beneficial bacteria.

The aim of the current research was to evaluate the effects of diverse environmental bacteria on the growth of the duckweed *L. minor*. Fifteen native bacterial communities collected from various aquatic environments were investigated for their effects on duckweed growth. Bacterial strains in communities that strongly enhanced or repressed the growth of duckweed were isolated for a more profound understanding of duckweed–microbe interactions.

## Results

### Effects of bacterial communities on duckweed growth

**Fig. 1:**
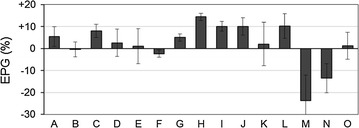
Effects on plant growth (EPGs) of bacterial communities collected from ponds or rivers. *A*–*O* indicate bacterial communities recovered from water samples. EPGs were evaluated based on the number of fronds after 7 days of cultivation compared to that of an aseptic control. There were 91.33 (±4.50), 85.67 (±2.05), and 81.33 (±6.60) fronds at the end of control experiments for bacterial communities *A*–*E*, *F*–*J*, and *K*–*O*. *Error bars* show the standard errors and include errors among treatments performed in triplicate and the control

**Fig. 2:**
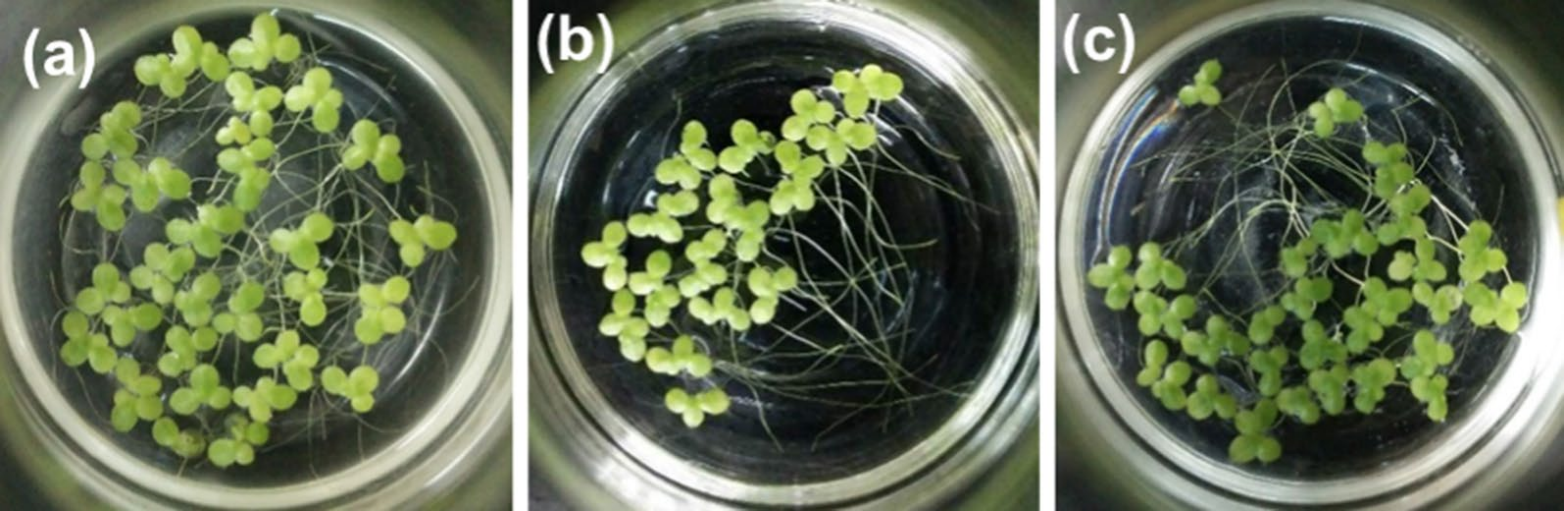
Images of *Lemna minor* after 7 days of cultivation with bacterial communities H (**a**) and M (**b**) and the aseptic control (**c**)

The growth of duckweed cultivated with fifteen environmental bacterial communities is shown in Fig. 1. Because all of the experiments were performed in the same axenic culture conditions, both promotive and inhibitory effects on duckweed growth should be a function of the bacterial communities. Many bacterial communities were found to have promotive or neutral effects on duckweed growth, with bacterial community H, which showed the greatest growth promotion, increasing the number of fronds by +14% over aseptically cultured *L. minor*. On the other hand, bacterial communities M and N decreased the number of duckweed fronds by −24 and −14%, respectively, compared to that of the controls. No remarkable difference in frond size, shape, color, or disease symptoms was observed in plants grown with the different bacterial communities in this series of experiments (Fig. 2).

### Isolation of bacterial strains from duckweed

From the plant bodies cultivated with bacterial communities H and M, which conferred the highest and lowest *L. minor* growth in the previous experiment, 10 and 12 morphologically distinct bacterial strains were isolated, respectively, and used for the further investigations. Table 1 shows the results of a BLAST search for the 16S rDNA sequences of all 22 isolates. All isolates showed at least 97% sequence identity with known strains. All isolates were found to be members of the alpha, beta, and gamma subclasses of *Proteobacteria*, except for H8, which belonged to the phylum *Actinobacteria*. In addition, both communities contained members of the order *Rhizobiales* (H1, H2, H5, M2), *Pseudomonadales* (H4, H6, M10, M12), *Burkholderiales* (H7, H9, M7, M8, M9), and *Sphingomonadales* (H10, M5, M11), which often comprise the large fraction of the rhizobacterial communities of terrestrial plants [31, 32].

**Table 1:** Nucleotide BLAST search of bacterial strains isolated from *Lemna minor* cultivated with communities H and M

Strain	Most similar species	Accession number of the closest match	Nucleotide similarity (%)
H1 (++)	*Starkeya koreensis*	NR_113962.1	98
H2 (−)	*Rhizobium daejeonense*	NR_114121.1	97
H3 (++)	*Aquitalea magnusonii*	NR_043475.1	99
H4 (+)	*Pseudomonas geniculata*	NR_024708.1	99
H5 (+/−)	*Methylobacterium fujisawaense*	NR_112232.1	99
H6 (−)	*Pseudomonas oryzihabitans*	NR_114041.1	99
H7 (+)	*Aquabacterium commune*	NR_024875.1	98
H8 (++)	*Leucobacter alluvii*	NR_042426.1	99
H9 (+/−)	*Acidovorax radicis*	NR_117776.1	99
H10 (+/−)	*Sphingomonas ursincola*	NR_119243.1	99
M1 (++)	*Azospirillum irakense*	NR_044949.1	99
M2 (++)	*Ensifer adhaerens*	NR_113893.1	98
M3 (− −)	*Acinetobacter ursingii*	NR_025392.1	100
M4 (+/−)	*Enterobacter ludwigii*	NR_042349.1	99
M5 (−−)	*Blastomonas natatoria*	NR_113794.1	99
M6 (−−)	*Asticcacaulis excentricus*	NR_074137.1	99
M7 (+/−)	*Ideonella dechloratans*	NR_026108.1	99
M8 (+)	*Aquincola tertiaricarbonis*	NR_043913.1	98
M9 (+/−)	*Rubrivivax gelatinosus*	NR_025841.1	98
M10 (+/−)	*Pseudomonas psychrotolerans*	NR_042191.1	99
M11 (+/−)	*Sphingomonas parapaucimobilis*	NR_113729.1	99
M12 (++)	*Pseudomonas otitidis*	NR_043289.1	99

**Fig. 3:**
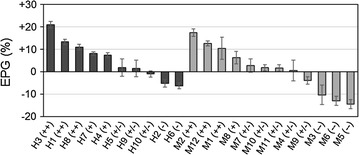
Effects on plant growth (EPGs) of single bacteria isolated from communities H (*black bars*) and M (*gray bars*). EPGs were evaluated by the change in dry weight of *Lemna minor* relative to that of the aseptic control, which had 119.67 (±5.19) fronds at the end. *Error bars* show the standard errors (*n* = 3)

Symbols in parentheses indicate the effects on plant growth of strains (Fig. 3): ++, greater than 10%; +, between +5 and +10%; +/−, between −5 and +5%; −, between −5 and −10%; −−, less than −10%

### Cultivation of *L. minor* with single bacterial isolates

A total of 22 isolates were evaluated for their effects on duckweed growth by co-culture with sterilized *L. minor*. In this experiment, frond numbers and dry weights were highly correlated (*r* = 0.93), so only the EPGs calculated from the dry weight were used in further analyses. As shown in Fig. 3, duckweed growth was affected both positively and negatively by the inoculation of isolates. The EPGs of members of bacterial community H varied from −6.3 to +21%, whereas in community M, they ranged from −14.4 to +17.5%. Bacterial strains were classified into five groups: those showed EPG values greater than 10% (++), between +5 and +10% (+), between −5 and +5% (+/−), between −5 and −10% (−), and less than −10% (−−). According to this classification scheme, there were 3, 2, 3, 2, and 0 isolates from community H, corresponding to (++), (+), (+/−), (−), and (−−), respectively, whereas there were 3, 1, 5, 0, and 3 isolates from community M. Bacterial communities H and M, which were associated the greatest and least growth in the first experiment, contained both promotive bacteria (++ or +) and inhibitory bacteria (− or −−). However, it is worth noting that inhibitory bacteria with EPG values less than −10% (−−) were isolated only from community M.

### Cultivation of *L. minor* with mixtures of two bacterial isolates

Based on the results of the previous experiment and ease of cultivation, promotive strains H1 (++), H3 (++), and M12 (++) and inhibitory strains H6 (−), M3 (−−), M5 (−−), and M6 (−−) were selected for further experiments, and all binary combinations of these isolates (_7_C_2_ = 21 patterns) were examined for their effects on duckweed growth. Simultaneously, single cultures of seven strains were tested again as positive controls. Because the frond number and dry weight showed a strong correlation (*r* = 0.95), only the dry weights were used to calculate EPGs (%). As shown in Table 4, all positive controls showed similar effects as those seen in the previous experiment, although the effects of H1 and H6 in this experiment were regarded as (+) and (−−), respectively. Generally, binary combinations of promotive bacteria showed promotive or neutral effects on duckweed growth, and combinations of inhibitory bacteria showed inhibitory effects. Combinations of promotive and inhibitory strains, however, resulted in both growth promotion and growth inhibition. Specifically, combinations of strain H3 (++) and an inhibitory bacterium tended to show promotive effects, whereas all combinations of strain H1 (+) and M12 (++) with inhibitory strains resulted in a negative effect.

### Evaluation of bacterial isolates for plant growth-affecting traits

The isolates were examined for traits that affect plant growth in a series of assays. The results for the 22 isolates are shown in Table 2. Of the 22 isolates, 10 could synthesize IAA, 11 could solubilize insoluble phosphate, and 12 could produce siderophores. In addition, 12 isolates were positive for hydrogen cyanide production, which is believed to be involved in plant growth inhibition. All of the isolates excepting for H2 (–) and H5 (+/−) exhibited at least one of these traits. Strains H3 (++), M1 (++), and M12 (++) were positive for all traits. The occurrence of these traits was similar between the isolates from communities H and M, except that the siderophore-producing isolates were more frequently found in community M than H. Table 3 shows the results of the multiple-way ANOVA test to detect the contribution of these traits to the effects on duckweed growth. Of the four traits, only phosphate solubilization was found to correlate (*p* < 0.05) with duckweed growth promotion, whereas the other traits correlated with neither growth promotion nor growth inhibition.

**Table 2:** Indole acetic acid (IAA) production, phosphate (P) P solubilization, siderophore production, and hydrogen cyanide (HCN) production by bacterial strains

Strain	IAA production	P solubilization	Siderophore production	HCN production
H1 (++)	−	+	−	−
H2 (−)	−	−	−	−
H3 (++)	+	+	+	+
H4 (+)	−	−	+	+
H5 (+/−)	−	−	−	−
H6 (−)	−	+	+	+
H7 (+)	+	+	−	−
H8 (++)	−	−	−	+
H9 (+/−)	+	−	−	+
H10 (+/−)	−	−	−	+
M1 (++)	+	+	+	+
M2 (++)	−	+	+	−
M3 (−−)	−	+	+	−
M4 (+/−)	−	+	+	+
M5 (−−)	−	−	−	−
M6 (−−)	+	−	+	+
M7 (+/−)	+	−	−	−
M8 (+)	+	−	−	−
M9 (+/−)	+	−	−	−
M10 (+/−)	−	+	+	+
M11 (+/−)	−	+	+	+
M12 (++)	+	+	+	+

Symbols in parentheses indicate the effects on plant growth of strains (Fig. 3): ++, greater than 10%; +, between +5 and +10%; +/−, between −5 and +5%; −, between −5 and −10%; −−, less than −10%

**Table 3:** The result of multiple-way analysis of variance (ANOVA)

Factor	Mean square	F value	Coefficients	Significance
IAA	141.79	1.986	4.88	0.177
P solub.	354.82	4.969	11.34	0.040
Sidero.	118.20	1.665	−7.41	0.216
HCN	39.29	0.550	3.12	0.468

*IAA* indole acetic acid production, *P solub.* phosphate solubilization, *sidero.* siderophore production, *HCN* hydrogen cyanide production were analyzed as factors related to the effects on plant growth (%) of 22 isolates

## Discussion

This study revealed that bacterial communities in freshwater environments can both enhance and repress the growth of duckweed *L. minor* (Fig. 1). The effects of bacterial communities on the 7-day growth of *L. minor* ranged from −24 to +14%, which indicates that a 1.5-fold difference in duckweed yield can be controlled by selecting a bacterial community. Approximately, the same change in relative growth rate of *L. minor* was observed with light intensities of 400 and 110 µmol/m^2^/s [20], and ammonium (as a sole nitrogen source) concentrations of 28 and 2 mg/L [33]. Considering these facts, environmental bacterial communities were shown to be a critical factor that affects duckweed growth, with effects that are comparable with other important environmental factors such as light and nutrients. Enhancement of crop yields by optimizing co-existing bacteria has long been a goal for sustainable agriculture. Here, our results show its feasibility, even for the hydroculture of duckweed. This strategy should be fascinating choice if attained, since co-existing bacteria can potentially be modulated with lower energy than light, nutrient, and temperature.

Although culture-dependent methods have clear limitations for analyzing bacterial communities, we believe that it is useful to isolate bacterial strains in order to characterize their functions, since it is expected that readily culturable bacteria comprise larger fraction in duckweed rhizoplane than that in other natural environments according to Matsuzawa et al. [28]. Moreover, to rationally design co-existent bacteria for enhanced duckweed biomass production, understanding which bacterial strains promote or inhibit duckweed growth is indispensable. In this study, we isolated and characterized representative bacterial strains from both promotive and inhibitory bacterial communities H and M, respectively (Fig. 3). Taxonomically, large part of the bacterial isolates belonged to taxa that are known inhabitants of the terrestrial plant rhizosphere. It might suggest that duckweed rhizobacteria share the same characteristics with those of terrestrial plants to a certain extent. Interestingly, both promotive and inhibitory communities contained bacterial strains that expressed promotive, inhibitory, and neutral effects on duckweed growth, and their isolation frequencies were not significantly different between the two communities. The only notable difference was that the activities of inhibitory bacterial strains isolated from the inhibitory community were stronger than those of the strains isolated from the promotive community. We conclude that promotive, inhibitory, and neutral bacteria are ubiquitous in duckweed-associated bacterial communities, and that the activities of these bacteria likely determine, synergistically, the net effect of a bacterial community on duckweed growth.

As far as we know, *Acinetobacter calcoaceticus* P23 [29] and *Exiguobacterium* sp. MH3 [30] are the only PGPB that have been reported for duckweed species. In this study, we discovered six new bacterial strains that promoted the growth of duckweed by more than 10% with 7 days of cultivation. Sequencing of 16S rRNA genes revealed that these strains belong to diverse genera that were different from previously isolated PGPB, suggesting that PGPB for duckweed are distributed across a wider range of taxa. Interestingly, strains M1 (++) and M12 (++) were identified as *Azospirillum* and fluorescent *Pseudomonas*, respectively, both of which are common PGPB for terrestrial plants, except for *Pseudomonas syringae*, which is a plant pathogen. On the other hand, the most efficient PGPB strain H3 (++) was identified as belonging to the genus *Aquitalea*, which has been discovered only in freshwater environments. Quisehuatl-Tepexicuapan et al. [34] isolated one strain of *Aquitalea* from the rhizoplane of duckweed *L. gibba*. Therefore, strain H3 may be a PGPB specific for aquatic plants, including duckweed, which has evolved in freshwater environments.

Bacterial strains that suppress the plant growth without any apparent pathogenic symptoms are known as plant growth-inhibiting bacteria (PGIB) or deleterious rhizobacteria (DRB) in the field of agriculture. Although these bacteria are difficult to detect, a number of studies indicate that PGIB and DRB can be regulated to improve crop production [35, 36] and to control weeds [37]. We isolated these bacteria for the first time from duckweed or aquatic plants in this study. Because these bacteria can significantly lower the efficiency of duckweed production, attention should be paid to PGIB, as well as PGPB. Interestingly, we found PGIB in the genus *Acinetobacter*, which is the same taxonomic group as that of the first PGPB identified in duckweed [29]. Therefore, culture-independent metagenomic analysis of the 16S rRNA gene is not sufficient to detect and distinguish between duckweed PGPB and PGIB in bacterial communities, and further isolation-based research, such as this study, will contribute to not only a deeper understanding of duckweed–microbe interactions but also the construction of a relevant bacterial database.

Many studies have been dedicated to elucidating the mechanisms by which PGPB affect plant growth [22]. In this study, we examined the correlation between EPGs assessed by co-cultivation and the presence of four physiological traits that are known to be associated with plant growth promotion or inhibition. Although many bacteria were found to have more than one of these traits (Table 2), no clear-cut correlation was found between the possession of these traits and duckweed growth promotion/inhibition effects of the bacterial strains in a multiple ANOVA analysis (Table 3). Therefore, multiple mechanisms, probably including unknown ones, are related to bacterial promotion/inhibition of duckweed growth. Among the four tested traits, only the ability to solubilize phosphate was shown to be slightly correlated with duckweed growth promotion. Although bacterial phosphate solubilization is widely recognized to contribute to phosphorus availability in soil environments [38], this result was unexpected, because all of the phosphorus was added in soluble form at the start of an experiment. However, it is possible that phosphate supply via degradation of dead bacterial cells, plant exudates, and phosphate salts formed in the medium was influenced by phosphate-solubilizing activity of duckweed-associated bacteria. Since aquatic environments also contain a variety of unavailable phosphorus [39], the effects of bacterial phosphate supply to plants should be evaluated in real hydroculture.

In contrast to our relatively substantial knowledge on the mechanisms of PGPB, reports on the mechanisms by which PGIB inhibit the growth of plants are quite limited. Cyanide production is virtually the only proposed mechanism with enough supporting data [40, 41], whereas other studies have suggested the benefit of hydrogen cyanide based on antifungal activity [42]. The current study did not show a significant correlation between cyanide production and plant growth. Further studies are required to understand duckweed growth inhibition associated with bacteria or bacterial community.

To better understand the complex effects of bacterial communities, effects of binary combinations of selected isolates on duckweed growth were tested as simple artificial bacterial community models. In contrast with results of a previous study conducted for terrestrial plant [43], synergistic effects were generally not observed with promotive–promotive or inhibitory–inhibitory bacterial combinations. Interestingly, the results of promotive–inhibitory bacterial combinations showed that promotive strains H1 (+) and M12 (++) were not effective in the presence of any of the inhibitory strains (Table 4). This suggests that not all PGPB are able to function in their native environments, and that inhibitory bacteria may have a stronger influence on the effects of the bacterial community as a whole. This observation shows the difficulty of using PGPB in non-sterilized conditions as reported in Liu et al. [44]. It also indicates that regulation of PGIB may be effective for maximizing PGPB activity in a bacterial community. In contrast to strains H1 (+) and M12 (++), promotive strain H3 (++) was less susceptible to the deleterious effects of inhibitory strains, and was found to exert a promotive effect or at least negate the inhibitory effects of other bacteria. From this point of view, strain *Aquitalea magnusonii* H3 can be regarded as a PGPB for potential use in open environments.

**Table 4:** The effects on plant growth (EPGs, %) based on dry weight of a mixed inoculation of two species of bacteria

	H3	M12	H1	H6	M6	M3	M5
H3	+24.8 (±1.7)						
M12	+15.5 (±3.2)	+11.6 (±4.2)					
H1	+23.7 (±2.6)	+1.8 (±3.6)	+5.1 (±1.9)				
H6	+6.7 (±4.5)	−7.4 (±2.2)	−11.3 (±5.1)	−10.6 (±2.5)			
M6	+15.7 (±1.6)	−16.7 (±4.0)	−7.0 (±5.3)	−7.7 (±1.9)	−13.2 (±4.1)		
M3	+3.1 (±2.9)	−15.9 (±3.1)	−3.8 (±5.4)	−20.2 (±3.6)	−15.0 (±6.5)	−14.0 (±3.0)	
M5	+10.9 (±2.7)	−19.5 (±3.5)	−12.0 (±4.7)	−10.5 (±4.4)	−6.8 (±3.9)	−13.9 (±3.4)	−19.2 (±3.1)

Rows and columns indicate the isolates used. The cells with rows and columns that indicate the same strain show the results of single inoculations as positive controls. There were 110.33 (±4.50) fronds at the end of control experiments. Values in parenthesis represent the mean ± standard error of the mean (*n* = 3)

There are many possible explanations for what determines the result of the conflicting effects of duckweed PGPB and PGIB described above. For example, competition between bacteria on root exudates and spaces, inactivation of promotive or inhibitory mechanisms, and masking effects are likely. Elucidating such bacterial interactions is an important next step for optimizing duckweed hydroculture systems via the design of beneficial bacterial communities. For this reason, bacterial strains obtained in this study may be useful as model PGPB and PGIB for duckweed.

## Conclusion

This study reported that (1) bacterial community strongly influences the production speed of duckweed biomass; (2) duckweed harbors bacteria which have promotive, neutral, or inhibitory effects for their growth; (3) promotive effects of PGPB strains can sustain or cannot sustain in the presence of other bacteria, depending on the kind of PGPB strain and some unknown mechanisms; and (4) many of isolates from duckweed-associated bacterial communities have some common characteristics in their taxa and ability to influence plant growth with terrestrial rhizobacteria. From these, it can be concluded that modulating bacterial community is the possible choice for improving biomass production from duckweed hydroculture. Further, it may be applicable to the other aquatic feedstocks such as water lettus, water hyacinth, and *Azolla* plants which have similar morphology to duckweed.

## Methods

### Plant material

Common duckweed (*Lemna minor*, RDSC #5512), obtained from a small pond in a botanical garden of Hokkaido University (Sapporo, Japan), was used in the experiments. The plants were sterilized by washing with 0.5% sodium hypochlorite for 7 min, followed by washing with sterilized water twice. The sterilized plants were successively cultured in flasks containing Hoagland medium (36.1 mg/L KNO_3_, 293 mg/L K_2_SO_4_, 3.87 mg/L NaH_2_PO_4_, 103 mg/L MgSO_4_·7H_2_O, 147 mg/L CaCl_2_·H_2_O, 3.33 mg/L FeSO_4_·7H_2_O, 0.95 mg/L H_3_BO_3_, 0.39 mg/L MnCl_2_·4H_2_O, 0.03 mg/L CuSO_4_·5H_2_O, 0.08 mg/L ZnSO_4_·7H_2_O, and 0.254 mg/L H_2_MoO_4_·4H_2_O; pH 7.0) in an incubation chamber at 28 °C, an irradiance of 80 µmol/m^2^/s, and a photoperiod of 16 h/8 h day/night.

### Cultivation of duckweed with environmental bacterial communities

Water samples were taken from the surfaces of 15 freshwater ponds and rivers located in the northern part of Osaka, Japan in August 24, 27, and 30 of 2015. Descriptions of sampled sites are shown in Additional file 1: Figure S1. The native bacterial communities in the samples were recovered and used for duckweed cultivation experiments as follows. First, coarse particles, including fungi and microalgae, were removed from the water samples using filters with a pore size of 3.0 µm (SSWP, MF-Millipore, Merck Millipore, Darmstadt, Germany), followed by centrifugation (10,000×*g*, 4 °C, 10 min) to collect bacterial cells from the native bacterial communities. The collected bacterial cells were washed twice with sterilized Hoagland medium and re-suspended in the original volume of Hoagland medium. Ten fronds of *L. minor* were transferred to flasks filled with 60 mL of the medium containing the bacteria and cultivated for 7 days in the above-mentioned conditions. During cultivation, duckweed growth was monitored by counting the frond number. The effects of the bacterial communities on duckweed growth were evaluated in comparison with growth of a control without introduced bacteria (sterile Hoagland medium).

### Isolation of bacterial strains attached to duckweed

At the end of 7 days of cultivation of duckweed with bacterial communities, whole plant bodies in each flask were collected and washed with 20 mL of sterilized 5 mg/L sodium tripolyphosphate (TPP). Then, the duckweed samples were homogenized in TPP using a BioMasher II (Nippi, Tokyo, Japan). The homogenates were spread onto solid 1:10 LB medium in TPP containing 1.5% agar and incubated at 28 °C for 7 days. All morphologically distinct colonies were picked and purified using the same medium.

### Identification of bacterial strains

Isolated bacterial strains were identified based on their 16S rRNA gene sequences. A single colony of each bacterial strain was picked and added to PCR reagent containing primers 27F (5′-AGAGTTTGATCTGGCTCAG-3′) [45] and 1392R (5′-ACGGGCGGTGTGTACA-3′) [46] and Ex Taq DNA polymerase (TaKaRa Bio Inc., Shiga, Japan). PCR amplification of the 16S rRNA gene fragments was performed as described previously [47] using a T100 Thermal Cycler (Bio-Rad Laboratories, Hercules, CA, USA). The amplicons were sequenced by Hokkaido System Science Co., Ltd (Hokkaido, Japan). The NCBI Nucleotide BLAST tool (http://blast.ncbi.nlm.nih.gov/Blast.cgi) was employed for taxonomic identification of strains H1–H10 and M1–12 using the obtained sequences as queries. The nucleotide sequences of the partial 16S rRNA gene from strains H1–H10 and M1–12 were submitted to the DNA Data Bank of Japan (DDBJ) under accession number LC191965–LC191986.

### Cultivation of duckweed with isolated bacterial strains

To cultivate bacterial isolates used in the experiments, a loop of bacterial colony was inoculated into 20 or 100 mL of liquid LB medium in a vial or flask that was held overnight at 28 °C with shaking at 120 rpm. Cells were harvested by centrifugation (10,000×*g*, 4 °C, 10 min), washed twice with sterilized Hoagland medium, and then re-suspended in the same medium with cells at an optical density at 600 nm (OD_600_) = 0.1. To allow bacterial strains to attach to the plants, aseptic *L. minor* were placed on each bacterial suspension for 24 h. Then, 10 duckweed fronds were transferred to a flask filled with fresh bacteria-free medium at the start of cultivation. This method minimized the effect of nutrient leakage from dead bacterial cells and enabled an evaluation of the direct physiological effects of bacteria on the duckweed [48]. After 7 days of cultivation, the number of duckweed fronds and dry weight (12 h drying at 80 °C) were measured. Cultivation experiments using a combination of two bacterial strains were performed using the same procedure, except that equal amounts (30 mL each) of two separately prepared bacterial suspensions (OD_600_ = 0.1) were mixed and allowed to attach to plants. Control experiments were performed using sterile Hoagland medium without the introduction of bacterial strains.

### Evaluation of bacterial isolates for traits that affect plant growth

#### Indole acetic acid (IAA) production

Indole acetic acid production in the presence of L-Trp was tested according to a method described by Orlando [49], with some modifications. First, a single bacterial colony was inoculated into a vial containing 20 mL of LB medium with 0.05% (w/v) of L-Trp. After 5 days of incubation with shaking (28 °C, 120 rpm), the culture was centrifuged (2000×*g*, 30 min, 24 °C), and 2 mL of the supernatant was added to 2 mL of Salkowski reagent (98 mL of 35% HClO_4_, 2 mL of 0.5 M FeCl_3_). Then, the mixture was placed at room temperature for 30 min for observation. Development of a pink color indicated the production of IAA.

#### Phosphate-solubilizing ability

The ability to solubilize insoluble phosphate was evaluated using Pikovskaya’s agar, which contains calcium phosphate as an insoluble phosphate [50]. Then, each bacterial colony was streaked onto an agar plate and incubated at 28 °C for 7 days. Results were considered positive when clear zones developed around a colony.

#### Siderophore production

Bacterial siderophore production was detected using the method of Schwyn and Neilands [51]. In this assay, each bacterial colony was streaked on a chrome azurol S (CAS) agar plate containing blue dye. Plates were incubated at 28 °C for 7 days and then examined for a yellow or orange halo around the colonies, which would indicate the production of a siderophore.

#### Hydrogen cyanide (HCN) production

The assay of bacterial cyanide production was performed according to a method described by Saber et al. [52]. In short, the bacterial strains were grown in 5 mL of LB medium in a test tube with Whatman No. 1 filter paper (GE Healthcare Life Science, Buckinghamshire, UK) soaked in cyanide reagent. Cyanide-producing bacteria were detected when the Whatman paper changed color from yellow to orange or red.

### Statistical analyses

Duckweed cultivation experiments were performed in triplicate for all treatments and controls. In all duckweed cultures, the effects on plant growth (*EPG*) of each bacterial community or each bacterial strain were calculated as follows:

$${\text{EPG }}({\% })\; = \;\frac{G\left( T \right) - G(C)}{G(C)}\; \times \;100,$$

where *G(T)* is the mean growth of duckweeds in the presence of microbes, which was evaluated by the frond number or dry weight of the duckweed after 7 days of cultivation, and *G(C)* is that in the aseptic controls. Here, the standard errors (SE) for EPG were calculated using the following formula:

$${\text{SE }}\left( {\text{EPG}} \right)\; = \;\frac{{\sqrt {{\text{SE}}\left( {G\left( T \right)} \right)^{2} + {\text{SE}}\left( {G\left( C \right)} \right)^{2} } }}{G(C)}\; \times \; 100.$$

Multiple-way analysis of variance (ANOVA) was performed to test whether bacterial IAA production, phosphate-solubilizing ability, siderophore production, or hydrogen cyanide production correlated with growth promotion or inhibition of *L. minor*. In this analysis, the results of four assays were treated as qualitative factors, and the EPG (%) based on dry weights in the duckweed cultivation experiments with single bacterial strains were used as the response variable. All statistical analyses were performed in R v3.2.3. (http://www.r-project.org).

## Additional file

**Additional file 1: Figure S1.** Locations and descriptions of water sampled sites.

## Abbreviations

PGPB: plant growth-promoting bacteria
BLAST: The Basic Local Alignment Search Tool
DNA: deoxyribonucleic acid
EPG: effects on plant growth
IAA: indole acetic acid
RNA: ribonucleic acid
PGIB: plant growth-inhibiting bacteria
DRB: deleterious rhizobacteria
NCBI: The National Center for Biotechnology Information
PCR: polymerase chain reaction
CAS: chrome azurol S
SE: standard error
