# Supplementary material for: Evaluation of environmental bacterial communities as a factor affecting the growth of duckweed Lemna minor
Source: Biotechnol Biofuels. 2017 Mar 10;10:62. doi: 10.1186/s13068-017-0746-8 (PMC5345205; doi:10.1186/s13068-017-0746-8)
Supplement: Supplementary file 1 — Additional file 1: Figure S1. Locations and descriptions of water sampled sites. [file 13068_2017_746_MOESM1_ESM.docx]

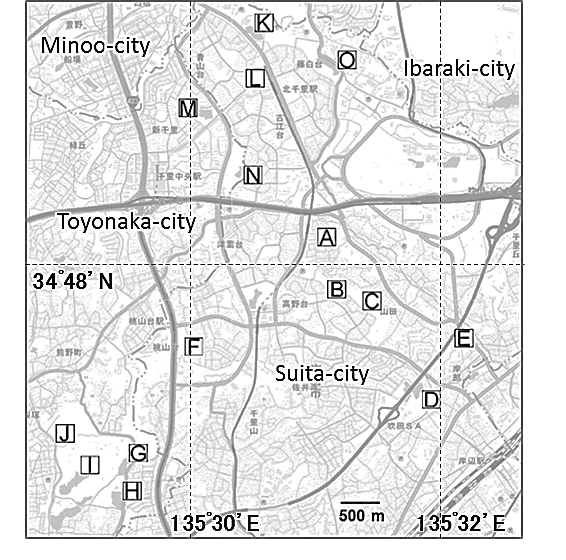
**Additional File 1**

**Figure S1.** Sampling locations: (A) small pond in residential area; (B) pond surrounded by trees; (C) small stream running through soil; (D) greenish pond in a park; (E) shallow stream in a residential area; (F) pond with many aquatic plants; (G) greenish swampy pond in a park; (H) small artificial pond with fountain; (I) pond in a park; (J) small river running through a residential area; (K) dirty pond in a park; (L) shallow, fast-running canal; (M) pond with fountain; (N) swampy pond surrounded by trees; (O) greenish pond in a residential area. Sampling dates (in 2015) were August 24 for samples (A)–(E), August 27 for samples (F)–(J), and August 30 for samples (K)–(O). The map was provided by the Geographical Information Authority of Japan (http://maps.gsi.go.jp/).
